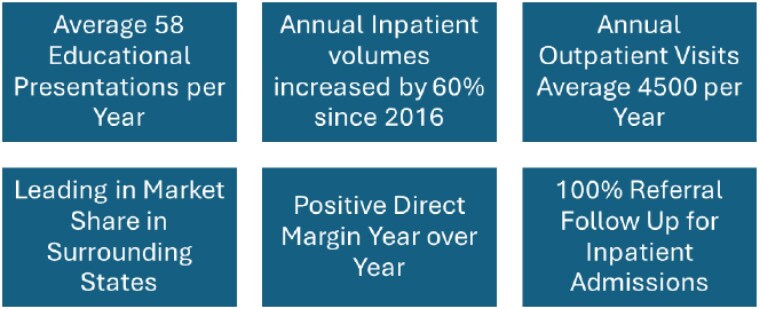# 616 Evaluating the Effectiveness of a Comprehensive Burn Outreach Program

**DOI:** 10.1093/jbcr/iraf019.245

**Published:** 2025-04-01

**Authors:** Heidi Altamirano, Mark Johnston

**Affiliations:** Regions Hospital; Regions Hospital

## Abstract

**Introduction:**

There are areas of the country with limited access to burn resources. Burn care is stressful in rural areas where providers infrequently see these specialized patients. Twelve years ago, a dedicated burn outreach and education position within a regional burn center was created. The primary goal of this role was to improve burn care in the region by providing access to education and resources for the rural providers. A secondary goal was to develop relationships to facilitate transfer to the burn center if needed. Specific activities of this role include travel to rural communities across the region to meet with EMS agencies and hospitals to provide resources and develop relationships, present educational content, participate in local and regional conferences, and follow up on referrals. An annual strategic plan is created to ensure the resources reach communities in need. The purpose of this work is to evaluate the effectiveness of the burn outreach role.

**Methods:**

A multimodal approach is used to evaluate the effectiveness of the burn outreach program. Annual inpatient and outpatient volumes, the number of educational presentations, evaluation of the annual outreach / education strategic plan, analysis of market share data, and regular review of burn profitability are included. These were evaluated over a period of years.

**Results:**

The primary goal of increasing access to burn care education and resources is met. The number of educational sessions to EMS agencies, rural hospitals, at conferences, and bringing Advanced Burn Life Support to the rural areas has increased now averaging nearly 60 sessions per year. The secondary goal of developing relationships is achieved. Market share shifted with the addition of this role, now being the leader in surrounding states. Inpatient admissions increased by 60% over the past eight years. The addition of referral follow- up has been a positive impact in relationship building with providers in rural areas and improved care by providing performance improvement-based feedback. The burn program has a positive direct margin, which is naturally a key outcome for administration.

**Conclusions:**

Thoughtfully creating a dedicated burn outreach and education role has been impactful in improving access to specialized burn education and resources. The development and execution of a targeted annual plan ensures that focus is on areas of need. The secondary benefits of increased inpatient and outpatient referrals, leading in market share, and a positive direct margin for the burn service line has been impactful for continued support.

**Applicability of Research to Practice:**

The role was developed for the right reasons with an experienced and passionate clinician, who is dedicated to burn care. Other centers would benefit from similar role to increase accessibility to specialized education and resources. Use of a multimodal approach to measure and analyze outcomes is applicable to other programmatic needs to gain and sustain administrative support.

**Funding for the Study:**

N/A